# Exploring Diversity among Norwegian *Borrelia* Strains Originating from *Ixodes ricinus* Ticks

**DOI:** 10.1155/2014/397143

**Published:** 2014-08-27

**Authors:** Ann-Kristin Tveten

**Affiliations:** Faculty of Life Sciences, Aalesund University College, 6025 Ålesund, Norway

## Abstract

Characterisation of *Borrelia* strains from *Ixodes ricinus* ticks is important in the epidemiological surveillance of vector-borne pathogens. Multilocus sequences analysis (MLSA) is a molecular genotyping tool with high discriminatory power that has been applied in evolutionary studies and for the characterisation of *Borrelia* genospecies. MLSA was used to study genetic variations in *Borrelia* strains isolated from *I. ricinus* ticks collected from the woodlands in Skodje. The results demonstrate that the 50 *Borrelia* strains were separated into 36 sequence types (STs) that were not previously represented in the MLST database. A distance matrix neighbour-joining tree (bootstrapped 500 iterations) showed four deeply branched clusters, and each deeply branched cluster represented one *Borrelia* genospecies. The mean pairwise genetic differences confirm the genospecies clustering. The combination of alleles separates the *Borrelia* strains from northwest Norway from the strains in the MLST database, thus identifying new STs. Although a highly divergent *B. afzelii* population could be expected, the heterogeneity among the *B. garinii* strains is more unusual. The present study indicates that the circulation of strains between migrating birds and stationary birds in this coastal region may play a role in the evolution of *B. garinii* strains.

## 1. Introduction

Ticks are considered important carriers of pathogenic microorganisms in the northern hemisphere [1], and* Ixodes ricinus* ticks are associated with a diverse microbiota [2].* Borrelia* spirochetes are one of the pathogens transmitted to humans by* I. ricinus* ticks [3], and* Borrelia burgdorferi* sensu lato (s.l.) represents a bacterial species complex that comprises several* Borrelia* genospecies associated with Lyme borreliosis (LB) [4].* Borrelia burgdorferi* sensu stricto (s.s.),* Borrelia afzelii,* and* Borrelia garinii* are causative agents of human LB in Norway [5], and the prevalence of LB is monitored by the Norwegian Surveillance System for Communicable Diseases (MSIS). A fourth* Borrelia* genospecies,* Borrelia valaisiana,* was discovered in Norwegian ticks in 2010 [5]. These four genospecies have been detected in* I. ricinus* ticks in northwest Norway [6].

During the recent years, several genotyping methods have been established for analysis of* Borrelia* to characterise strains and determine the phylogenetic relationships between strains [7–11]. Multilocus sequence typing (MLST) is a molecular typing tool that is used to characterise pathogenic microorganisms [12], and MLST can be used as a tool for the epidemiological surveillance and tracking of infectious diseases [13]. MLST, as defined by Urwin and Maiden, is a genotyping tool based on the sequences of housekeeping genes that evolve slowly. The sequences used for MLST are approximately 400–500 bp in size and are located throughout the genome to avoid bias [13]. Multilocus sequence analysis (MLSA) applies MLST to characterise closely related species using a distance-based procedure for phylogenetic characterisation, and it includes pairwise genetic similarities. MLST/MLSA have been applied in population studies and for analysis of* Borrelia* species in geographic areas and for evolutionary studies and characterisation of new* Borrelia* species. While some MLST/MLSA schemes combine housekeeping genes with hypervariable regions and noncoding loci [9–11], the MLST scheme published in 2008 by Margos et al. is based on housekeeping genes that fulfill the strict criteria defined by Urwin and Maiden [7, 13]. The* Borrelia* MLST scheme is based on amplification, sequencing, and bioinformatic analysis of internal fragments of eight housekeeping genes (*clpA, clpX, nifS, pepX, pyrG, rplB, recG,* and* uvrA*) [7].

The* Borrelia* MLST scheme is available through the MLST network (http://www.mlst.net/). The MLST network enables easy access to sequences from all over the world [14]. Recently, studies of phylogenetic relationship of* Borrelia* genospecies using MLSA have resulted in the definition of two new* Borrelia *species,* Borrelia bavariensis* sp. [15] and* Borrelia kurtenbachii* [8]. These new findings demonstrate the high discriminatory power that MLST/MLSA schemes provide. Rudenko et al. utilised MLST to describe recombination at the* nifS* locus among* B. burgdorferi* s.s. and* Borrelia americana* strains. Their findings indicated that, to a degree, strain diversity is influenced by the host [16]. The aim of this study is to utilise MLSA to characterise* Borrelia* strains isolated from* I. ricinus* ticks collected from the fauna in northwest Norway. Multilocus sequence analysis of Norwegian strains could provide knowledge about the strain diversity among* Borrelia *strains found in this region and describe their evolution compared to European strains.

## 2. Materials and Methods

### 2.1. Samples

**Figure 1:**
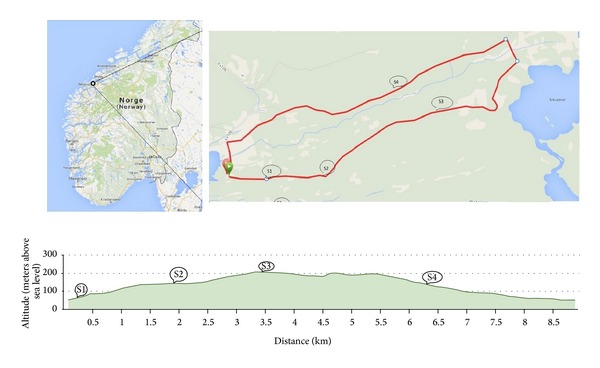
Map of the collection site. The map indicates the location site in northwest Norway and the tracking route used. The altitude of each collection site and the distance between the sites are indicated in the bottom part of the map.

DNA from questing* I. ricinus* ticks collected in 2012 (531 nymphs/71 adults) and 2013 (539 nymphs/64 adults) (Figure 1) was isolated, and the DNA samples were analysed by qPCR to detect the presence of* Borrelia* genospecies as previously described [6]. A total of 86 samples (79 nymphs/7 adults) were positive for* Borrelia* in 2012, and 89 samples (83 nymphs/8 adults) were positive for* Borrelia* in 2013. Multilocus sequence analysis was performed using the MLST scheme developed by Margos et al. A total of 50 samples were amplified across all eight housekeeping genes (*clpA, clpX, nifS, pepX, pyrG, recG, rplB,* and* uvrA*) [7] and were submitted for sequencing. The 50 strains used in this study are listed in Table 1.

### 2.2. Amplification and Sequencing

The amplification of each housekeeping gene was carried out in a 15 *µ*L reaction volume containing 7.5 *µ*L Mastermix 2.0x SYBR GREEN (Applied Biosystems), 0.75 *µ*L of each primer (10 *µ*M), 3.00 *µ*L ddH_2_O, and 3.0 *µ*L template DNA. Amplification of the eight housekeeping genes (*clpA, clpX, nifS, pepX, pyrG, recG, rplB,* and* uvrA*) was performed with primers and nested touchdown PCR conditions for* clpA, nifS, pepX, pyrG, recG,* and* uvrA* as previously described [7, 8]. Amplification of* clpX* and* rplB* was performed with primers described by Margos et al. combined with a nested touchdown PCR composed of 1 cycle of denaturation (10 min, 95°C), followed by 9 cycles of denaturation (30 sec, 95°C), touchdown annealing (30 sec, 58°C–50°C), and extension (1 min, 72°C) and 30 cycles of denaturation (30 sec, 95°C), annealing (30 sec, 50°C), and extension (1 min, 72°C) with a final cycle of extension (10 min, 72°C). Gel electrophoresis was used to confirm the size and approximate concentration of the PCR products for each amplified gene. Gel electrophoresis was performed with a 2% Tris-acetate EDTA buffered (TBE) gel followed by staining with GelRed (Affymetrix, Santa Clara, USA).

The PCR products were purified in a two-step process by ExoSAP-IT (Affymetrix, Santa Clara, USA) according to the manufacturers' protocol. The sequencing reaction was carried out in a 10 *µ*L reaction volume containing 1 *µ*L Big Dye Terminator 3.1 (Applied Biosystems, Carlsbad, USA), 1 *µ*L 5x Sequencing Buffer (Applied Biosystems, Carlsbad, USA), 0.32 *µ*L primer (10 *µ*M), 4.68 *µ*L ddH_2_O, and 3 *µ*L template DNA. Amplification was performed in a 2720 thermal cycler (Applied Biosystems, Carlsbad, USA) with 1 cycle of denaturation (6 min, 96°C), followed by 25 cycles of denaturation (10 sec, 96°C), annealing (5 sec, 50°C), and extension (4 min, 60°C). Finally, 10 *µ*L ddH_2_O was added to each well upon completion of the sequencing reaction, and the samples were stored at –20°C until sequencing. Sequencing was performed by Eurofins MWG Operon (Germany).

### 2.3. Alignment and Multilocus Sequence Analysis

The sequences for each housekeeping gene were aligned by the MUSCLE algorithm and concatenated in MEGA 5.1 [17]. The sequences were analysed with BLAST to confirm the correct* Borrelia* genospecies [18]. Each locus and the concatenated sequence of all eight loci were analysed. The MEGA 5.1 software was used to draw a distance matrix neighbour-joining tree (bootstrapped 500 iterations) with the concatenated DNA sequences. Sequences from* B. duttonii* were added from the BLAST database [18] to root the neighbour-joining tree.* B. duttonii* is a* Borrelia* species that causes relapsing fever [19]. Sequences from four reference species from the MLST database were included in the analysis,* B. burgdorferi* s.s. (B31),* B. afzelii* (VS461),* B. valaisiana* (70865B), and* B. garinii* (20047). To compare allele sequences and sequences types (STs) and to identify identical sequences, the nonredundant database (NRDB) written by Warren Gish at Washington University was used (http://pubmlst.org/analysis/). All known alleles and STs were retrieved from the* Borrelia* MLST database for comparison (http://borrelia.mlst.net/). STs that were not found in the MLST database were submitted to the MLST database for proper numbering. Statistical analysis was performed with MEGA 5.1. The mean pairwise genetic difference (within and between groups) was calculated using the Kimura 2-parameter model. Average nonsynonymous substitutions and synonymous substitutions (d*N*/d*S*) were calculated using the modified Nei-Gojobori method (Jukes-Cantor). The number of polymorphic sites (PS) and the nucleotide diversity (*π*) were calculated using Tajima's test of neutrality. The index of association, both standard and classic, was calculated using START2 [20].

To compare the relationship between Norwegian* Borrelia* strains and European* Borrelia* strains a goeBURST minimum spanning tree was generated using PHYLOViZ 1.0 software [21].

## 3. Results

**Figure 2:**
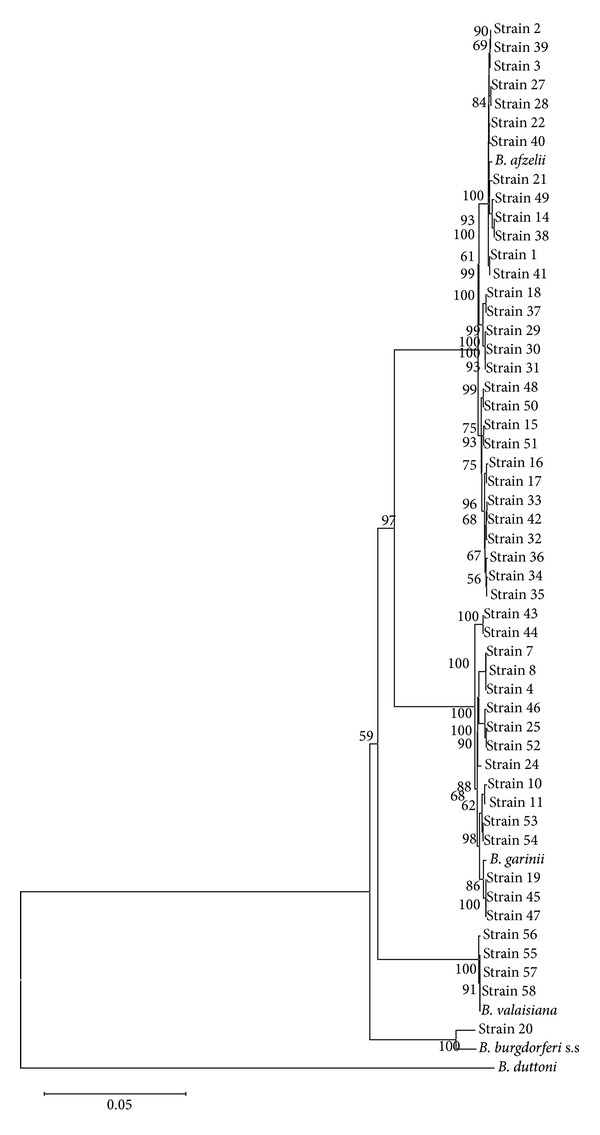
Description of clades based on the neighbour-joining tree (bootstrapped 500 iterations) calculated based on concatenated DNA sequences showing the relationship between* B. burgdorferi* s.l. strains.

**Figure 3:**
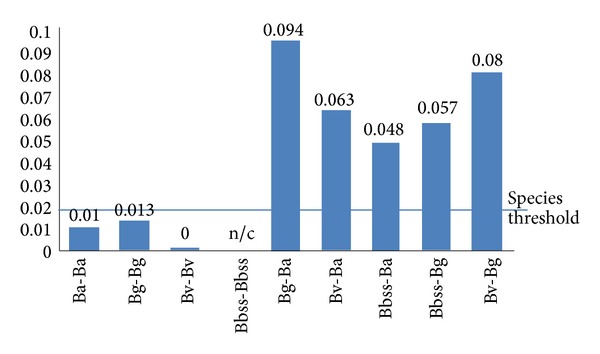
Mean pairwise difference within and between* Borrelia* genospecies. The line indicates species threshold (0.017) as defined by Margos et al. [15].

A total of 50 strains were sequenced across all eight loci for the present study. A distance matrix neighbour-joining tree (bootstrapped 500 iterations) (Figure 2) demonstrated that the 50 strains were separated into 36 sequence types (STs) (Table 1) that were not previously found in the MLST database. The neighbour-joining tree formed four deeply branched clusters, and, between the four clusters, there were no shared alleles. All strains cluster together with their respective reference species and contain combinations of alleles that are new entries into the MLST database. The mean pairwise genetic difference confirms the genospecies clustering (Figure 3) based on the genospecies threshold (0.017) as defined by Margos et al. [15].  Table 2 summarises the characteristics of each of the eight housekeeping genes included in the multilocus sequencing analysis of* Borrelia* strains.

**Figure 4:**
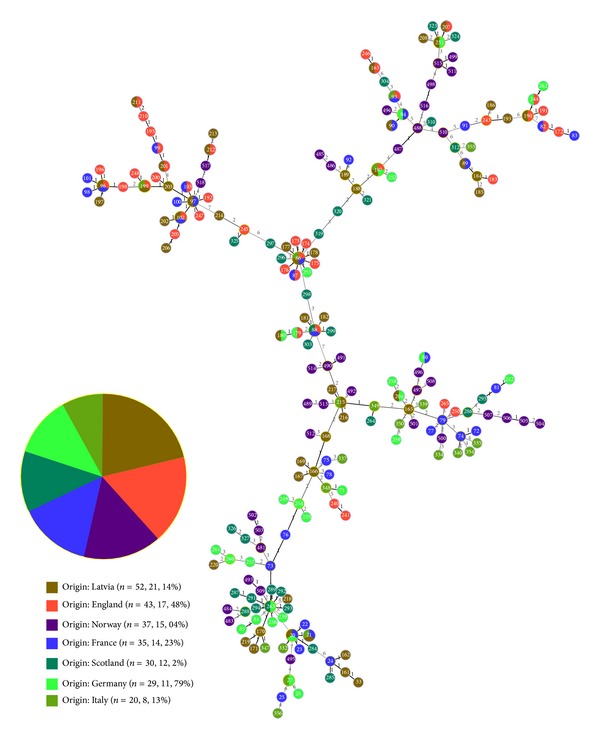
goeBURST. The goeBURST is coloured based on the geographic origin of each ST. Multiple colours indicated multiple origins.

The comparative goeBURST analysis (level 8 grouping) displays the relationship between the Norwegian strains and European strains (Figure 4). The goeBURST analysis shows that the Norwegian* Borrelia* population is the most closely related to the Latvian and French populations. The dataset contains 175 unique STs and information for approximately 246 strains. A goeBURST analysis was performed at the SLV (single locus variant) level by dividing the dataset into 32 groups with 2 or more STs and 64 singletons. The largest clonal complex 0 (CC0) has 13 unique STs and represents 33 strains. CC0 represents strains identified as* B. valaisiana* and contains 6 SLVs. All of the SLVs share the* pepX, pyrG, rplB,* and* uvrA* alleles. In CC1 (9 STs, 22 strains), the predicted ancestral genotype is ST 86, with 8 SLVs representing 10 strains; thus this is the highest ranking ancestral genotype. All SLVs have the same* clpA, clpX,* and* rplB* alleles. A goeBURST analysis conducted at the DLV (double locus variant) level divided the dataset into 54 CCs consisting of 26 groups with 2 or more STs and 28 singletons. A goeBURST analysis conducted at the TLV (three loci variant) level divided the dataset into 33 CCs consisting of 21 groups with 2 or more STs and 11 singletons. A level 4 grouping was required for* B. valaisiana* to be represented by one CC in the goeBURST analysis, and a level 5 grouping was required for* B. afzelii* to be represented by one CC. A level 7 grouping was required for* B. garinii* and* B. burgdorferi* s.s. to be represented by one CC.

The Norwegian STs are characterised as both SLVs and DLVs. The Norwegian strains are spread throughout the goeBURST tree and are clustered together with their respective genospecies from other European countries. Four branches are formed from the primary founder. These four branches contain STs related to the four genospecies,* B. garinii, B. afzelii, B. valaisiana,* and* B. burgdorferi* s.s. The highest ranking ancestral genotype, ST 86, is a* B. garinii* strain from Latvia.

## 4. Discussion

Northwest Norway is characterised by geographical features such as islands, several deep fjords, and high mountains. The landscape shows signs of overgrowth and little maintenance of the vegetation, thus providing suitable growth conditions for diverse wildlife. More than 4000 different Animalia species have been observed in this region [22]. The region has mild winters and humid summers, creating good conditions for* Ixodes ricinus* ticks.* Borrelia* spirochetes are maintained in nature by the circulation between the vector and the host, and the wide range of available hosts may influence the diversity among strains of* Borrelia* [23].* Borrelia* spirochetes have an obligate parasitic lifestyle, and their circulation depends on an interaction between reservoir hosts and vectors [24]. Birds are reservoir hosts for* B. garinii* while* B. afzelii* are usually associated with rodents [15, 25]. Evolution and a wide range of determinants that affect reservoir hosts and vectors are likely to influence the diversity among strains differently [23, 24, 26]. Reservoir hosts and vectors are submitted to a number of factors such as climate, nutrition, and pollution that have different impacts depending on the geographic locations [23, 24]. In addition to the influence that reservoir hosts provide, the complex microbial communities associated with* I. ricinus* ticks also subject* Borrelia* spirochetes to microbial competition and natural selection. These microbial communities are composed of bacteria, endosymbionts, viruses, and protozoa from the tick's natural habitat and hosts, and, therefore, they vary between different geographical locations [2, 27, 28].

Multilocus sequence analysis of the* Borrelia* strains found in* I. ricinus* ticks from this region shows signs of high diversity. The majority of the strains are* B. afzelii* and* B. garinii*, the most prevalent genospecies within this region [6]. Fifty* Borrelia* strains were separated into 36 STs, and the genetic relationship between the strains was visualised in a neighbour-joining tree. The first deeply branched cluster contains strains identified as* B. afzelii,* and 29 strains were separated into 22 STs. These 29* B. afzelii* strains form a highly structured population consisting of STs that have not been previously described in the MLST database. In 2011, Vollmer et al. demonstrated that there is limited movement of* B. afzelii* strains between geographic locations and that only two* B. afzelii* STs have been identified in more than one geographic location [22, 29].* B. afzelii* strains are described as heterogenic, and a comparative goeBURST analysis of strains from Scotland and strains from England showed that there is a limited movement of strains between the north and south in the United Kingdom [24, 29]. The dispersal of* Borrelia* spirochetes is linked to the movement of their hosts.* B. afzelii* is associated with small rodent hosts with a limited movement pattern, thus creating a limited dispersal of* B. afzelii* strains [30]. All strains included in the present study were collected from four sites within one geographically defined area (Figure 1). Some of the* B. afzelii* strains were identified in more than one sample, but they were always within the collection site.

The second deeply branched cluster contains four* B. valaisiana* strains divided into 2 STs that represent new entries into the MLST database. The third deeply branched cluster contains 16* B. garinii* strains divided into 11 STs that are new entries into the MLST database. Both* B. valaisiana* and* B. garinii* are bird-associated genospecies, and previous studies have found that these two* Borrelia* genospecies have sequence types that have been identified in different geographic locations throughout Europe [29]. Vollmer et al. reported that* B. valaisiana* strains are not completely homogenised within Europe while* B. garinii* shows signs of being a mixed population across central Europe [29]. Migrating birds play an important role in the dispersal of* B. garinii* strains between geographic locations. Kjelland et al. studied the prevalence of* Borrelia* infection in 31 species of migrating birds and 6 species of resident birds in southern Norway.* Borrelia* infection was found in all bird species [31]. Studies also suggest that* B. garinii* strains have the ability to circulate both in terrestrial cycles and in seabirds with their associated tick* Ixodes uriae* [32–34]. A study by Comstedt et al. from 2011 determined that* B. garinii* was found in Norwegian populations of seabirds and* I. uriae* [35]. Sequencing of the intergenic spacer (IGS) from* B. garinii* strains has indicated that the seabird-associated* B. garinii* strains are more diverse than the terrestrial* B. garinii* strains [36]. All strains included in the present study are collected from a geographic location with a high number of observed bird species. In Møre and Romsdal county, 290 bird species have been observed in 2013 by ornithologist [37]. These birds represent a variety of different reservoir hosts for* B. garinii*, and the nesting on the island provides a possibility of transferring* B. garinii* into fauna, inducing circulation between migrating and stationary hosts. The high number of migrating birds along the coastal area of northwest Norway enables introduction of* B. garinii* strains from multiple different geographic origins and multiple reservoir hosts and the process of circulation between reservoir hosts has been suggested to play a role in the genetic evolution of* Borrelia* genospecies [29]. Some* B. garinii* strains from the present study are DLVs and TLVs of the strains from central Europe, while others have levels 4 and 5 connections to the central European STs. Within the Norwegian* B. garinii* strains, some are SLVs and DLVs, and some are more distantly related. This may indicate that the different strains have been introduced into the fauna by migrating birds at different times and that some strains might have been maintained by stationary hosts for some time. Calculation of the nucleotide diversity (*π* value) reveals that the *π* values were at least two times greater for* B. garinii* than for* B. afzelii*. Assuming a similar generation time between* B. garinii* and* B. afzelii*, the different reservoir hosts might have influenced the evolutionary rate differently between genospecies [26]. The limited geographic dispersal of* B. afzelii* strains has allowed them to evolve differently between geographic locations, but the nucleotide diversity within a geographic location is relatively low.* B. garinii* associated with migratory birds is subjected to a higher degree of ecological determinants, and the nucleotide diversity may reflect that strains have been introduced to the fauna at different times.

The fourth deep branched cluster contains one* B. burgdorferi* s.s. isolate and its associated reference species. The* B. burgdorferi* s.s. strain was isolated from an adult female tick. The low prevalence of this genospecies [6], in combination with certain challenges associated with amplification across all eight loci, caused this strain to be the only* B. burgdorferi* s.s. included in this study.

The number of polymorphic sites (PS) and the nucleotide diversity (*π*) were calculated to further describe the* Borrelia* strains. A sequence analysis was conducted to identify point mutations found in multiple strains. These point mutations have a minor contribution to the genetic diversity between concatenated DNA sequences from northwest Norway compared to other worldwide strains. Most of the point mutations are silent or missense mutations with little or no effect on the genetic function. The ratio between nonsynonymous substitutions and synonymous substitutions (d*N*/d*S*) < 1 indicates that the number of synonymous substitutions is higher than the number of nonsynonymous substitutions. This indicates that the genes are not under selective pressure and are suitable for MLSA analysis. The index of association (*I*_*A*_) describes the influence of recombination in a population and was calculated in two ways: using the classical method defined by Smith et al. and using the standard method defined by Haubold and Hudson [38, 39]. The *I*_*A*_ would be significant for a clonal population. The calculated values for this population were *I*_*A*classic_ = 2.2054 and *I*_*A*standard_ = 0.3151, and significant linkage disequilibrium was detected. Estimation of the index of association indicated that the population is clonal and that recombination has not played an important role in the evolution of this population.

Multilocus sequencing analysis has provided knowledge about the genetic variations between* Borrelia* genospecies originating from* I. ricinus* ticks from northwest Norway. The combinations of alleles separate these strains from other strains in the MLST database. This is the first multilocus sequence analysis of* Borrelia* strains identified from Norwegian* I. ricinus* ticks, and the 50* Borrelia* strains from Norway represent a large contribution to the MLST database. This enabled comparative studies between Norwegian and European strains, thus creating a better understanding of the strain development of* Borrelia* genospecies in different geographic locations in Europe. The high level of divergence in the* B. afzelii* population was expected, but the divergence within the* B. garinii* population differs from previous MLST/MLSA studies. Migrating birds are constantly introducing new strains to the fauna along the coastline in northwest Norway, and circulation between migrating and stationary hosts would influence the strain diversity. Some of the* B. garinii* strains show signs of being newly introduced to the fauna while others have most likely been maintained in stationary hosts for a longer period of time.

## Figures and Tables

**Table 1:** Allele and sequence types (STs).

Source	Species	*clpA *	*clpX *	*nifS *	*pepX *	*pyrG *	*recG *	*rplB *	*uvrA *	ST
*I. ricinus *	*B. afzelii *	109	24	23	85	22	29	23	29	481
*I. ricinus *	*B. afzelii *	36	24	96	31	119	29	23	28	483
*I. ricinus *	*B. afzelii *	36	24	96	31	22	30	23	28	484
*I. ricinus *	*B. garinii *	46	30	33	42	29	88	79	36	485
*I. ricinus *	*B. garinii *	47	30	33	42	111	88	79	36	486
*I. ricinus *	*B. garinii *	47	30	33	42	111	88	79	36	486
*I. ricinus *	*B. garinii *	46	30	33	38	32	76	32	36	487
*I. ricinus *	*B. garinii *	46	30	29	38	32	76	32	36	488
*I. ricinus *	*B. afzelii *	37	24	23	35	96	30	99	142	489
*I. ricinus *	*B. afzelii *	51	82	23	86	85	27	28	29	490
*I. ricinus *	*B. afzelii *	51	82	23	86	85	27	29	29	491
*I. ricinus *	*B. afzelii *	172	24	23	85	85	27	29	29	492
*I. ricinus *	*B. afzelii *	37	135	24	87	22	29	23	28	493
*I. ricinus *	*B. garinii *	45	30	32	96	29	38	30	36	494
*I. ricinus *	*B. burgdorferi *s.s.	14	8	14	10	10	137	9	129	495
*I. ricinus *	*B. afzelii *	36	24	23	35	26	33	23	28	496
*I. ricinus *	*B. afzelii *	36	24	23	32	22	33	23	28	497
*I. ricinus *	*B. garinii *	46	30	29	96	32	76	79	33	498
*I. ricinus *	*B. garinii *	95	74	34	96	32	88	79	85	499
*I. ricinus *	*B. afzelii *	36	82	23	32	119	27	23	28	500
*I. ricinus *	*B. afzelii *	36	82	23	87	22	27	23	28	501
*I. ricinus *	*B. afzelii *	109	24	23	35	22	98	23	29	502
*I. ricinus *	*B. afzelii *	109	24	23	85	22	98	23	29	503
*I. ricinus *	*B. afzelii *	36	24	24	87	22	29	29	30	504
*I. ricinus *	*B. afzelii *	36	24	23	31	22	29	29	30	505
*I. ricinus *	*B. afzelii *	36	24	23	31	20	29	29	30	506
*I. ricinus *	*B. afzelii *	36	24	23	32	20	29	29	28	507
*I. ricinus *	*B. afzelii *	36	24	96	32	92	33	29	28	508
*I. ricinus *	*B. afzelii *	37	135	24	87	22	29	23	28	493
*I. ricinus *	*B. afzelii *	37	24	23	35	96	30	99	142	489
*I. ricinus *	*B. afzelii *	36	24	96	31	119	29	23	28	483
*I. ricinus *	*B. afzelii *	37	135	24	87	22	29	23	28	509
*I. ricinus *	*B. afzelii *	109	24	23	85	22	29	23	29	481
*I. ricinus *	*B. afzelii *	36	24	23	31	22	29	29	30	505
*I. ricinus *	*B. garinii *	46	74	29	38	35	40	32	33	510
*I. ricinus *	*B. garinii *	46	74	29	38	35	40	32	33	510
*I. ricinus *	*B. garinii *	45	30	32	96	29	38	30	36	494
*I. ricinus *	*B. garinii *	95	74	34	96	34	76	79	85	511
*I. ricinus *	*B. garinii *	45	30	32	96	29	38	30	36	494
*I. ricinus *	*B. afzelii *	36	24	23	35	85	29	28	29	512
*I. ricinus *	*B. afzelii *	51	24	23	86	85	30	99	142	513
*I. ricinus *	*B. afzelii *	36	24	23	35	85	29	28	29	512
*I. ricinus *	*B. afzelii *	51	82	23	86	119	29	28	29	514
*I. ricinus *	*B. garinii *	95	74	34	96	32	76	79	85	515
*I. ricinus *	*B. garinii *	46	30	29	96	32	76	32	33	516
*I. ricinus *	*B. garinii *	46	30	29	96	32	76	32	33	516
*I. ricinus *	*B. valaisiana *	96	76	36	98	84	44	78	86	517
*I. ricinus *	*B. valaisiana *	50	76	36	98	38	44	78	86	518
*I. ricinus *	*B. valaisiana *	96	76	36	98	84	44	78	86	517
*I. ricinus *	*B. valaisiana *	96	76	36	98	84	44	78	86	517

**Table 2:** Characteristics of each housekeeping gene.

Species	Gene	Size (bp)	Avg. G+C content %	PS (%)^a^	*π* ^b^	d*N*/d*S* ^c^
*B. burgdorferi *s.s.	*clpA *	579	52.2	—^d^	—	—
*B. afzelii *				0,69	0,001759	0,001810
*B. garinii *				1,73	0,005484	0,005205
*B. valaisiana *				0,69	0,003454	0,003768

*B. burgdorferi *s.s.	*clpX *	624	56.8	—	—	—
*B. afzelii *				0,48	0,001255	0,00122
*B. garinii *				6,08	0,02191	0,001835
*B. valaisiana *				—^e^	—	—

*B. burgdorferi *s.s.	*nifS *	564	55.8	—	—	—
*B. afzelii *				0,36	0,00087	0,00090
*B. garinii *				1,06	0,0054	0,00434
*B. valaisiana *				—	—	—

*B. burgdorferi *s.s.	*pepX *	570	55.6	—	—	—
*B. afzelii *				0,88	0,00328	0,00223
*B. garinii *				1,23	0,00484	0,00501
*B. valaisiana *				—	—	—

*B. burgdorferi *s.s.	*pyrG *	603	51.0	—	—	—
*B. afzelii *				0,83	0,00240	0,00211
*B. garinii *				1,49	0,00805	0,00609
*B. valaisiana *				1,32	0,0073	0,00637

*B. burgdorferi *s.s.	*recG *	651	53.0	—	—	—
*B. afzelii *				0,62	0,00209	0,00157
*B. garinii *				1,54	0,00545	0,00463
*B. valaisiana *				—	—	—

*B. burgdorferi *s.s.	*rplB *	624	56.9	—	—	—
*B. afzelii *				0,61	0,00261	0,00143
*B. garinii *				1,28	0,00669	0,00386
*B. valaisiana *				—	—	—

*B. burgdorferi *s.s.	*uvrA *	570	54.4	—	—	—
*B. afzelii *				0,88	0,00234	0,00223
*B. garinii *				1,05	0,00335	0,003172
*B. valaisiana *				—	—	—

^a^Percent polymorphic sites (PS (%)).

^b^Nucleotide diversity (*π*).

^c^Average nonsynonymous substitutions versus synonymous substitutions (d*N*/d*S*).

^d^It was not possible to perform statistics with only one isolate in this group.

^e^It was not possible to perform statistics with only one allele variant.
